# Supplementary material for: The genetic organization of longitudinal subcortical volumetric change is stable throughout the lifespan
Source: eLife. 2021 Jun 28;10:e66466. doi: 10.7554/eLife.66466 (PMC8260220; doi:10.7554/eLife.66466)
Supplement: Source code 1. [file elife-66466-code1.zip › Cluster tests.docx]

**Intra- vs. extra-cluster correlation tests**

Cluster Tests

library(readxl)
library(tidyverse)

We define the clusters in a list.

clusters <- list(
 Cluster1 = c("Bilatv3rdVentricle", "Bilatv4thVentricle", "BilatLatVentricle",
 "BilatInfLatVentricle"),
 Cluster2 = c("BilatBrainStem", "BilatCerebellumWM", "BilatCerebellumCortex",
 "BilatThalamus", "BilatHippocampus", "BilatCerebralWM"),
 Cluster3 = c("BilatCerebralCortex", "BilatPutamen", "BilatAmygdala",
 "BilatAccumbens"),
 Cluster4 = "BilatCaudate",
 Cluster5 = "BilatPallidum"
)


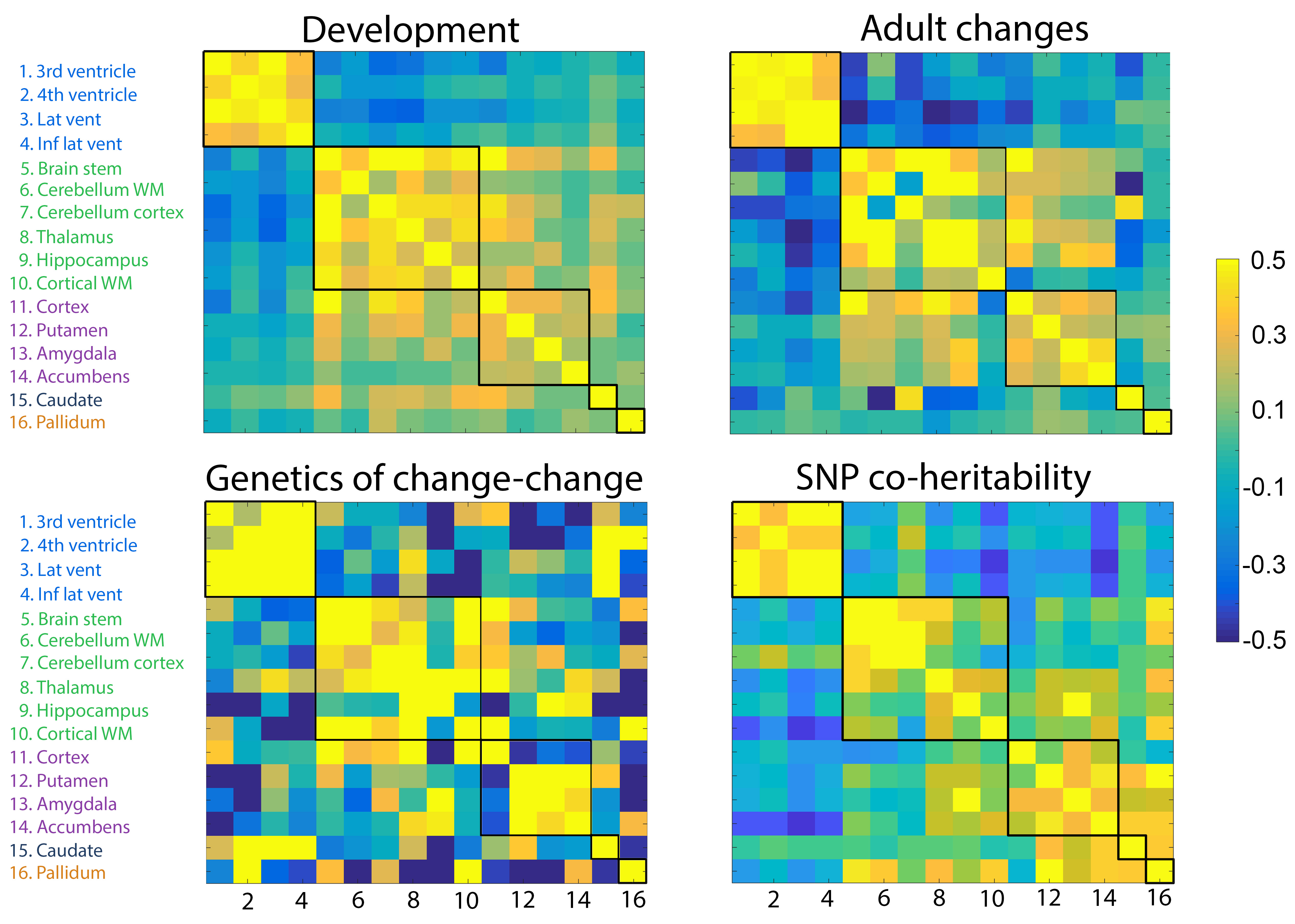


Function for testing correlations.

test_correlations <- function(dataset){
 imap(clusters, function(cl, nm){
 if(length(cl) > 1){
 correlations <- dataset %>%
 filter(Structure %in% cl) %>% ## Subset to the right rows
 pivot_longer(cols = -Structure, names_to = "Structure2",
 values_to = "correlation") %>% # Reshape data
 filter(Structure != Structure2) %>% # Remove diagonal
 mutate( # Define whether it is within or outside cluster
 within_cluster = Structure2 %in% cl
 ) %>%
 select(within_cluster, correlation) %>%
 group_by(within_cluster) %>%
 summarise(correlations = list(correlation))

 within_correlations <- correlations %>%
 filter(within_cluster) %>%
 pull(correlations) %>%
 unlist()

 between_correlations <- correlations %>%
 filter(!within_cluster) %>%
 pull(correlations) %>%
 unlist()

 cat("Testing in ", nm, "\n")

 test <- t.test(
 x = within_correlations,
 y = between_correlations,
 alternative = "greater"
 )

 print(test)
 cat("\n\n")

 return(test$p.value)
 } else {
 cat(nm, "has only a single member. No test performed.\n\n")
 return(NA_real_)
 }

 })
}

## UK Biobank

Read in the data.

ukb <- read_excel("UKB_GeneticCorr_Change_symmetric.xlsx") %>%
 rename(Structure = "...1") %>%
 rename_at(vars(-Structure), ~ paste0("Bilat", .)) %>%
 mutate(Structure = paste0("Bilat", Structure))

## New names:
## * `` -> ...1

Perform two-group t-tests in each cluster, testing if the correlation is larger in within than between. Information about the test is printed below.

ukb_tests <- test_correlations(ukb)

## Testing in Cluster1
##
## Welch Two Sample t-test
##
## data: within_correlations and between_correlations
## t = 12.148, df = 15.745, p-value = 1.04e-9
## alternative hypothesis: true difference in means is greater than 0
## 95 percent confidence interval:
## 0.5237763 Inf
## sample estimates:
## mean of x mean of y
## 0.5092477 -1.1025374
##
##
##
## Testing in Cluster2
##
## Welch Two Sample t-test
##
## data: within_correlations and between_correlations
## t = 5.805, df = 60.243, p-value = 1.283e-07
## alternative hypothesis: true difference in means is greater than 0
## 95 percent confidence interval:
## 0.1745083 Inf
## sample estimates:
## mean of x mean of y
## 0.29909213 0.05407273
##
##
##
## Testing in Cluster3
##
## Welch Two Sample t-test
##
## data: within_correlations and between_correlations
## t = 5.5518, df = 41.305, p-value = 9.169e-07
## alternative hypothesis: true difference in means is greater than 0
## 95 percent confidence interval:
## 0.157487 Inf
## sample estimates:
## mean of x mean of y
## 0.28288517 0.05691312
##
##
##
## Cluster4 has only a single member. No test performed.
##
## Cluster5 has only a single member. No test performed.

## VETSA / Genetics of change-change

Read in the data.

# VETSA
vetsa <- read_delim("Subcort_GeneticCorr_Change_ed.csv", delim = ";",
 locale = locale(decimal_mark = ",")) %>%
 rename(Structure = X1)

## Warning: Missing column names filled in: 'X1' [1]

## Parsed with column specification:
## cols(
## X1 = col_character(),
## BilatThalamus = col_double(),
## BilatCaudate = col_double(),
## BilatPutamen = col_double(),
## BilatPallidum = col_double(),
## BilatHippocampus = col_double(),
## BilatAmygdala = col_double(),
## BilatAccumbens = col_double(),
## BilatInfLatVentricle = col_double(),
## BilatLatVentricle = col_double(),
## BilatCerebralWM = col_double(),
## BilatCerebralCortex = col_double(),
## BilatCerebellumCortex = col_double(),
## BilatCerebellumWM = col_double(),
## Bilatv3rdVentricle = col_double(),
## Bilatv4thVentricle = col_double(),
## BilatBrainStem = col_double()
## )

Perform two-group t-tests in each cluster, testing if the correlation is larger in within than between. Information about the test is printed below.

vetsa_tests <- test_correlations(vetsa)

## Testing in Cluster1
##
## Welch Two Sample t-test
##
## data: within_correlations and between_correlations
## t = 8.2789, df = 36.711, p-value = 3.21e-10
## alternative hypothesis: true difference in means is greater than 0
## 95 percent confidence interval:
## 0.5412866 Inf
## sample estimates:
## mean of x mean of y
## 0.5668262 -0.1130322
##
##
##
## Testing in Cluster2
##
## Welch Two Sample t-test
##
## data: within_correlations and between_correlations
## t = 5.5531, df = 67.32, p-value = 2.578e-07
## alternative hypothesis: true difference in means is greater than 0
## 95 percent confidence interval:
## 0.3130808 Inf
## sample estimates:
## mean of x mean of y
## 0.39946907 -0.04800671
##
##
##
## Testing in Cluster3
##
## Welch Two Sample t-test
##
## data: within_correlations and between_correlations
## t = 0.98457, df = 13.426, p-value = 0.1711
## alternative hypothesis: true difference in means is greater than 0
## 95 percent confidence interval:
## -0.1400012 Inf
## sample estimates:
## mean of x mean of y
## 0.15888474 -0.01736666
##
##
##
## Cluster4 has only a single member. No test performed.
##
## Cluster5 has only a single member. No test performed.

## Lifebrain

Translation of names for Lifebrain.

translate_lifebrain <- function(old_name){
 lifebrain_translation <- list(
 Accumbensarea_APC = "BilatAccumbens",
 Amygdala_APC = "BilatAmygdala",
 BrainStem_APC = "BilatBrainStem",
 Caudate_APC = "BilatCaudate",
 CerebellumCortex_APC = "BilatCerebellumCortex",
 CerebellumWhiteMatter_APC = "BilatCerebellumWM",
 CortexVol_APC = "BilatCerebralCortex",
 CerebralWhiteMatterVol_APC = "BilatCerebralWM",
 Hippocampus_APC = "BilatHippocampus",
 InfLatVent_APC = "BilatInfLatVentricle",
 LateralVentricle_APC = "BilatLatVentricle",
 Pallidum_APC = "BilatPallidum",
 Putamen_APC = "BilatPutamen",
 Thalamus_APC = "BilatThalamus",
 X3rdVentricle_APC = "Bilatv3rdVentricle",
 X4thVentricle_APC = "Bilatv4thVentricle"
 )

 if(!old_name %in% names(lifebrain_translation)){
 stop(old_name, "not found")
 }

 lifebrain_translation[[old_name]]
}

Read in the data and translate.

lifebrain <- read_excel("Lifebrain_change_change_n836.xlsx") %>%
 rename(Structure = "...1") %>%
 rename_at(vars(-Structure), ~ map_chr(., translate_lifebrain)) %>%
 mutate(Structure = map_chr(Structure, translate_lifebrain))

## New names:
## * `` -> ...1

Perform two-group t-tests in each cluster, testing if the correlation is larger in within than between. Information about the test is printed below.

lifebrain_tests <- test_correlations(lifebrain)

## Testing in Cluster1
##
## Welch Two Sample t-test
##
## data: within_correlations and between_correlations
## t = 11.283, df = 15.321, p-value = 3.985e-09
## alternative hypothesis: true difference in means is greater than 0
## 95 percent confidence interval:
## 0.5347269 Inf
## sample estimates:
## mean of x mean of y
## 0.4726826 -0.1602484
##
##
##
## Testing in Cluster2
##
## Welch Two Sample t-test
##
## data: within_correlations and between_correlations
## t = 3.7976, df = 50.148, p-value = 0.0001978
## alternative hypothesis: true difference in means is greater than 0
## 95 percent confidence interval:
## 0.1045314 Inf
## sample estimates:
## mean of x mean of y
## 0.1363119 -0.0507810
##
##
##
## Testing in Cluster3
##
## Welch Two Sample t-test
##
## data: within_correlations and between_correlations
## t = 2.9104, df = 23.332, p-value = 0.003904
## alternative hypothesis: true difference in means is greater than 0
## 95 percent confidence interval:
## 0.06001752 Inf
## sample estimates:
## mean of x mean of y
## 0.139147746 -0.006711137
##
##
##
## Cluster4 has only a single member. No test performed.
##
## Cluster5 has only a single member. No test performed.

## LCBC

This correlation matrix is sorted after the order of regions appearing in the figure above. The order is as follows.

(lcbc_names <- unname(unlist(clusters)))

## [1] "Bilatv3rdVentricle" "Bilatv4thVentricle" "BilatLatVentricle"
## [4] "BilatInfLatVentricle" "BilatBrainStem" "BilatCerebellumWM"
## [7] "BilatCerebellumCortex" "BilatThalamus" "BilatHippocampus"
## [10] "BilatCerebralWM" "BilatCerebralCortex" "BilatPutamen"
## [13] "BilatAmygdala" "BilatAccumbens" "BilatCaudate"
## [16] "BilatPallidum"

Read in the data.

lcbc <- read_delim("corrmat_adults_sorted.txt", delim = ",",
 col_names = lcbc_names) %>%
 mutate(Structure = lcbc_names)

## Parsed with column specification:
## cols(
## Bilatv3rdVentricle = col_double(),
## Bilatv4thVentricle = col_double(),
## BilatLatVentricle = col_double(),
## BilatInfLatVentricle = col_double(),
## BilatBrainStem = col_double(),
## BilatCerebellumWM = col_double(),
## BilatCerebellumCortex = col_double(),
## BilatThalamus = col_double(),
## BilatHippocampus = col_double(),
## BilatCerebralWM = col_double(),
## BilatCerebralCortex = col_double(),
## BilatPutamen = col_double(),
## BilatAmygdala = col_double(),
## BilatAccumbens = col_double(),
## BilatCaudate = col_double(),
## BilatPallidum = col_double()
## )

Perform two-group t-tests in each cluster, testing if the correlation is larger in within than between. Information about the test is printed below.

lcbc_tests <- test_correlations(lcbc)

## Testing in Cluster1
##
## Welch Two Sample t-test
##
## data: within_correlations and between_correlations
## t = 17.141, df = 33.991, p-value < 2.2e-16
## alternative hypothesis: true difference in means is greater than 0
## 95 percent confidence interval:
## 0.5812216 Inf
## sample estimates:
## mean of x mean of y
## 0.4456900 -0.1991452
##
##
##
## Testing in Cluster2
##
## Welch Two Sample t-test
##
## data: within_correlations and between_correlations
## t = 6.2174, df = 71.134, p-value = 1.534e-08
## alternative hypothesis: true difference in means is greater than 0
## 95 percent confidence interval:
## 0.2575573 Inf
## sample estimates:
## mean of x mean of y
## 0.29512893 -0.05674885
##
##
##
## Testing in Cluster3
##
## Welch Two Sample t-test
##
## data: within_correlations and between_correlations
## t = 6.1225, df = 50.806, p-value = 6.677e-08
## alternative hypothesis: true difference in means is greater than 0
## 95 percent confidence interval:
## 0.166707 Inf
## sample estimates:
## mean of x mean of y
## 0.28430333 0.05479108
##
##
##
## Cluster4 has only a single member. No test performed.
##
## Cluster5 has only a single member. No test performed.

## Summary

The table below summarizes the p-values for each sample.

bind_rows(
 UKB = ukb_tests,
 VETSA = vetsa_tests,
 Lifebrain = lifebrain_tests,
 LCBC = lcbc_tests,
 .id = "Dataset"
) %>%
 knitr::kable()

| Dataset | Cluster1 | Cluster2 | Cluster3 | Cluster4 | Cluster5 |
| --- | --- | --- | --- | --- | --- |
| UKB | 0 | 0.0000001 | 0.0000009 | NA | NA |
| VETSA | 0 | 0.0000003 | 0.1711224 | NA | NA |
| Lifebrain | 0 | 0.0001978 | 0.0039035 | NA | NA |
| LCBC | 0 | 0.0000000 | 0.0000001 | NA | NA |
