# Supplementary material for: The genetic organization of longitudinal subcortical volumetric change is stable throughout the lifespan
Source: eLife. 2021 Jun 28;10:e66466. doi: 10.7554/eLife.66466 (PMC8260220; doi:10.7554/eLife.66466)
Supplement: Supplementary file 1. [file elife-66466-supp1.docx]

**Supplementary file**

**UKB SNP co-heritability**

| Trait1 | Trait2 | rg | se | P | p < .05 |
| --- | --- | --- | --- | --- | --- |
| 3rd_ventricle | 4th_ventricle | 0.369 | 0.026 | 0.00E+00 | * |
| 3rd_ventricle | LatVent | 0.611 | 0.022 | 0.00E+00 | * |
| 3rd_ventricle | Brain_stem | -0.102 | 0.026 | 5.29E-05 | * |
| 3rd_ventricle | Cerebellum_wm | -0.095 | 0.031 | 1.30E-03 | * |
| 3rd_ventricle | Cerebellum_cortex | 0.161 | 0.028 | 1.39E-09 | * |
| 3rd_ventricle | Thalamus | -0.209 | 0.029 | 6.43E-11 | * |
| 3rd_ventricle | Hippocampus | -0.089 | 0.031 | 3.23E-03 | * |
| 3rd_ventricle | Cortical_WM | -0.248 | 0.028 | 7.77E-16 | * |
| 3rd_ventricle | Cortex | -0.039 | 0.036 | 1.43E-01 |  |
| 3rd_ventricle | Putamen | -0.079 | 0.029 | 3.96E-03 | * |
| 3rd_ventricle | Amygdala | -0.123 | 0.034 | 2.67E-04 | * |
| 3rd_ventricle | Accumbens | -0.358 | 0.029 | 0.00E+00 | * |
| 3rd_ventricle | Caudate | 0.065 | 0.028 | 1.07E-02 | * |
| 3rd_ventricle | Pallidum | -0.173 | 0.029 | 3.51E-09 | * |
| 3rd_ventricle | InfLatVent | 0.585 | 0.029 | 0.00E+00 | * |
| 4th_ventricle | LatVent | 0.360 | 0.026 | 0.00E+00 | * |
| 4th_ventricle | Brain_stem | 0.051 | 0.021 | 7.39E-03 | * |
| 4th_ventricle | Cerebellum_wm | -0.045 | 0.025 | 3.57E-02 | * |
| 4th_ventricle | Cerebellum_cortex | 0.228 | 0.021 | 0.00E+00 | * |
| 4th_ventricle | Thalamus | -0.040 | 0.025 | 5.52E-02 | * |
| 4th_ventricle | Hippocampus | -0.012 | 0.025 | 3.23E-01 |  |
| 4th_ventricle | Cortical_WM | -0.140 | 0.024 | 3.27E-09 | * |
| 4th_ventricle | Cortex | -0.027 | 0.029 | 1.70E-01 |  |
| 4th_ventricle | Putamen | -0.026 | 0.024 | 1.29E-01 |  |
| 4th_ventricle | Amygdala | -0.027 | 0.028 | 1.65E-01 |  |
| 4th_ventricle | Accumbens | -0.136 | 0.027 | 4.36E-07 | * |
| 4th_ventricle | Caudate | 0.079 | 0.023 | 2.35E-04 | * |
| 4th_ventricle | Pallidum | -0.059 | 0.024 | 6.46E-03 | * |
| 4th_ventricle | InfLatVent | 0.371 | 0.032 | 0.00E+00 | * |
| LatVent | Brain_stem | -0.179 | 0.026 | 6.47E-12 | * |
| LatVent | Cerebellum_wm | -0.133 | 0.030 | 1.47E-05 | * |
| LatVent | Cerebellum_cortex | 0.076 | 0.028 | 2.67E-03 | * |
| LatVent | Thalamus | -0.296 | 0.028 | 0.00E+00 | * |
| LatVent | Hippocampus | -0.273 | 0.028 | 2.78E-16 | * |
| LatVent | Cortical_WM | -0.328 | 0.026 | 0.00E+00 | * |
| LatVent | Cortex | -0.111 | 0.035 | 1.59E-03 | * |
| LatVent | Putamen | -0.186 | 0.028 | 2.31E-10 | * |
| LatVent | Amygdala | -0.178 | 0.033 | 3.90E-07 | * |
| LatVent | Accumbens | -0.426 | 0.027 | 0.00E+00 | * |
| LatVent | Caudate | 0.123 | 0.028 | 7.17E-06 | * |
| LatVent | Pallidum | -0.171 | 0.029 | 5.03E-09 | * |
| LatVent | InfLatVent | 0.759 | 0.021 | 0.00E+00 | * |
| Brain_stem | Cerebellum_wm | 0.741 | 0.012 | 0.00E+00 | * |
| Brain_stem | Cerebellum_cortex | 0.417 | 0.016 | 0.00E+00 | * |
| Brain_stem | Thalamus | 0.400 | 0.019 | 0.00E+00 | * |
| Brain_stem | Hippocampus | 0.183 | 0.022 | 4.44E-16 | * |
| Brain_stem | Cortical_WM | 0.320 | 0.019 | 0.00E+00 | * |
| Brain_stem | Cortex | -0.123 | 0.026 | 7.60E-07 | * |
| Brain_stem | Putamen | 0.182 | 0.020 | 0.00E+00 | * |
| Brain_stem | Amygdala | 0.064 | 0.025 | 4.82E-03 | * |
| Brain_stem | Accumbens | 0.162 | 0.024 | 2.31E-11 | * |
| Brain_stem | Caudate | 0.086 | 0.020 | 9.27E-06 | * |
| Brain_stem | Pallidum | 0.463 | 0.017 | 0.00E+00 | * |
| Brain_stem | InfLatVent | -0.073 | 0.032 | 1.27E-02 | * |
| Cerebellum_wm | Cerebellum_cortex | 0.554 | 0.017 | 0.00E+00 | * |
| Cerebellum_wm | Thalamus | 0.300 | 0.024 | 0.00E+00 | * |
| Cerebellum_wm | Hippocampus | 0.144 | 0.026 | 6.47E-08 | * |
| Cerebellum_wm | Cortical_WM | 0.350 | 0.022 | 0.00E+00 | * |
| Cerebellum_wm | Cortex | -0.117 | 0.032 | 7.17E-05 | * |
| Cerebellum_wm | Putamen | 0.130 | 0.024 | 8.46E-08 | * |
| Cerebellum_wm | Amygdala | 0.013 | 0.029 | 3.31E-01 |  |
| Cerebellum_wm | Accumbens | 0.150 | 0.028 | 1.86E-07 | * |
| Cerebellum_wm | Caudate | 0.031 | 0.024 | 9.91E-02 | * |
| Cerebellum_wm | Pallidum | 0.381 | 0.022 | 0.00E+00 | * |
| Cerebellum_wm | InfLatVent | -0.127 | 0.037 | 5.60E-04 | * |
| Cerebellum_cortex | Thalamus | 0.161 | 0.022 | 1.93E-12 | * |
| Cerebellum_cortex | Hippocampus | 0.099 | 0.023 | 1.43E-05 | * |
| Cerebellum_cortex | Cortical_WM | -0.027 | 0.023 | 1.13E-01 |  |
| Cerebellum_cortex | Cortex | -0.037 | 0.027 | 8.06E-02 |  |
| Cerebellum_cortex | Putamen | 0.078 | 0.021 | 1.52E-04 | * |
| Cerebellum_cortex | Amygdala | 0.032 | 0.025 | 1.02E-01 |  |
| Cerebellum_cortex | Accumbens | 0.005 | 0.025 | 4.20E-01 | * |
| Cerebellum_cortex | Caudate | 0.095 | 0.021 | 2.13E-06 | * |
| Cerebellum_cortex | Pallidum | 0.146 | 0.021 | 1.22E-11 | * |
| Cerebellum_cortex | InfLatVent | 0.104 | 0.034 | 9.61E-04 | * |
| Thalamus | Hippocampus | 0.297 | 0.024 | 0.00E+00 | * |
| Thalamus | Cortical_WM | 0.350 | 0.022 | 0.00E+00 | * |
| Thalamus | Cortex | 0.123 | 0.030 | 5.34E-05 | * |
| Thalamus | Putamen | 0.242 | 0.023 | 0.00E+00 | * |
| Thalamus | Amygdala | 0.202 | 0.027 | 1.57E-11 | * |
| Thalamus | Accumbens | 0.252 | 0.027 | 5.55E-17 | * |
| Thalamus | Caudate | 0.119 | 0.024 | 3.59E-07 | * |
| Thalamus | Pallidum | 0.372 | 0.022 | 0.00E+00 | * |
| Thalamus | InfLatVent | -0.228 | 0.036 | 3.86E-09 | * |
| Hippocampus | Cortical_WM | 0.198 | 0.024 | 4.18E-14 | * |
| Hippocampus | Cortex | 0.168 | 0.030 | 1.13E-07 | * |
| Hippocampus | Putamen | 0.238 | 0.023 | 0.00E+00 | * |
| Hippocampus | Amygdala | 0.527 | 0.021 | 0.00E+00 | * |
| Hippocampus | Accumbens | 0.232 | 0.027 | 2.85E-14 | * |
| Hippocampus | Caudate | 0.116 | 0.024 | 8.12E-07 | * |
| Hippocampus | Pallidum | 0.218 | 0.024 | 0.00E+00 | * |
| Hippocampus | InfLatVent | -0.091 | 0.039 | 1.23E-02 | * |
| Cortical_WM | Cortex | 0.050 | 0.030 | 5.01E-02 | * |
| Cortical_WM | Putamen | 0.205 | 0.023 | 0.00E+00 | * |
| Cortical_WM | Amygdala | 0.124 | 0.028 | 1.02E-05 | * |
| Cortical_WM | Accumbens | 0.275 | 0.026 | 0.00E+00 |  |
| Cortical_WM | Caudate | 0.017 | 0.023 | 2.27E-01 |  |
| Cortical_WM | Pallidum | 0.405 | 0.020 | 0.00E+00 | * |
| Cortical_WM | InfLatVent | -0.297 | 0.034 | 4.22E-15 | * |
| Cortex | Putamen | 0.142 | 0.028 | 4.56E-07 | * |
| Cortex | Amygdala | 0.290 | 0.030 | 1.11E-16 | * |
| Cortex | Accumbens | 0.222 | 0.031 | 1.19E-10 | * |
| Cortex | Caudate | 0.101 | 0.027 | 1.31E-04 | * |
| Cortex | Pallidum | -0.081 | 0.029 | 2.68E-03 | * |
| Cortex | InfLatVent | -0.142 | 0.043 | 9.82E-04 | * |
| Putamen | Amygdala | 0.264 | 0.025 | 0.00E+00 | * |
| Putamen | Accumbens | 0.417 | 0.023 | 0.00E+00 | * |
| Putamen | Caudate | 0.334 | 0.020 | 0.00E+00 | * |
| Putamen | Pallidum | 0.576 | 0.017 | 0.00E+00 | * |
| Putamen | InfLatVent | -0.106 | 0.036 | 1.76E-03 | * |
| Amygdala | Accumbens | 0.361 | 0.027 | 0.00E+00 | * |
| Amygdala | Caudate | 0.188 | 0.026 | 7.56E-13 | * |
| Amygdala | Pallidum | 0.193 | 0.027 | 3.06E-12 | * |
| Amygdala | InfLatVent | -0.099 | 0.042 | 1.16E-02 | * |
| Accumbens | Caudate | 0.327 | 0.025 | 0.00E+00 | * |
| Accumbens | Pallidum | 0.357 | 0.025 | 0.00E+00 | * |
| Accumbens | InfLatVent | -0.377 | 0.036 | 0.00E+00 | * |
| Caudate | Pallidum | 0.361 | 0.020 | 0.00E+00 | * |
| Caudate | InfLatVent | 0.117 | 0.035 | 4.13E-04 | * |
| Pallidum | InfLatVent | -0.078 | 0.036 | 1.64E-02 | * |

**SNP based heritability estimates** Pairwise co-heritability between brain structures derived from 38.127participants from UKB. Age, sex, ICV and genetic ancestry (the first 10 components) were used as covariates. * p < .05 (uncorrected)
