# Supplementary material for: The genetic organization of longitudinal subcortical volumetric change is stable throughout the lifespan
Source: eLife. 2021 Jun 28;10:e66466. doi: 10.7554/eLife.66466 (PMC8260220; doi:10.7554/eLife.66466)
Supplement: Transparent reporting form [file elife-66466-transrepform.docx]

***eLife’s* transparent reporting form**

We encourage authors to provide detailed information *within their submission* to facilitate the interpretation and replication of experiments. Authors can upload supporting documentation to indicate the use of appropriate reporting guidelines for health-related research (see [EQUATOR Network](http://www.equator-network.org/%20)), life science research (see the [BioSharing Information Resource](https://biosharing.org/)), or the [ARRIVE guidelines](http://www.plosbiology.org/article/info:doi/10.1371/journal.pbio.1000412) for reporting work involving animal research. Where applicable, authors should refer to any relevant reporting standards documents in this form.

If you have any questions, please consult our Journal Policies and/or contact us: [editorial@elifesciences.org](mailto:editorial@elifesciences.org).

**Sample-size estimation**

- You should state whether an appropriate sample size was computed when the study was being designed
- You should state the statistical method of sample size computation and any required assumptions
- If no explicit power analysis was used, you should describe how you decided what sample (replicate) size (number) to use

Please outline where this information can be found within the submission (e.g., sections or figure legends), or explain why this information doesn’t apply to your submission:

No explicit power analysis was performed when the study was designed. We included available longitudinal data from Center for Lifespan Changes in Brain and Cognition ([www.oslobrains.no](http://www.oslobrains.no)). To reduce the risk of non-replicable results, analyses were run on independent longitudinal datasets from the Lifebrain consortium, VETSA (Vietnam Era Twin Study of Aging) and UK Biobank, and the results formally compared using Mantel tests. Although power cannot be assessed retrospectively, the observed effect sizes and precision estimates suggest that power was excellent.

**Replicates**

- You should report how often each experiment was performed
- You should include a definition of biological versus technical replication
- The data obtained should be provided and sufficient information should be provided to indicate the number of independent biological and/or technical replicates
- If you encountered any outliers, you should describe how these were handled
- Criteria for exclusion/inclusion of data should be clearly stated
- High-throughput sequence data should be uploaded before submission, with a private link for reviewers provided (these are available from both GEO and ArrayExpress)

Please outline where this information can be found within the submission (e.g., sections or figure legends), or explain why this information doesn’t apply to your submission:

There were no experiments performed Criteria for inclusion/ exclusion are described in the manuscript in the section “Sample” in the Materials and Methods. Moore details are provided in Supplemental Information in the section “1. Sample”

**Statistical reporting**

- Statistical analysis methods should be described and justified
- Raw data should be presented in figures whenever informative to do so (typically when N per group is less than 10)
- For each experiment, you should identify the statistical tests used, exact values of N, definitions of center, methods of multiple test correction, and dispersion and precision measures (e.g., mean, median, SD, SEM, confidence intervals; and, for the major substantive results, a measure of effect size (e.g., Pearson's r, Cohen's d)
- Report exact p-values wherever possible alongside the summary statistics and 95% confidence intervals. These should be reported for all key questions and not only when the p-value is less than 0.05.

Please outline where this information can be found within the submission (e.g., sections or figure legends), or explain why this information doesn’t apply to your submission:

The statistical analysis methods are described in the main manuscript in the sections “Experimental Design and Statistical Analysis” and “Genetic correlations”. In addition, details are provided in Supplemental Information in the sections “6. Heritability estimates for change-change in VETSA” and “8. Intra- vs. extra-cluster correlation tests”. R- and matlab code used to generate the statistical results are provided with the manuscript.

(For large datasets, or papers with a very large number of statistical tests, you may upload a single table file with tests, Ns, etc., with reference to sections in the manuscript.)

**Group allocation**

- Indicate how samples were allocated into experimental groups (in the case of clinical studies, please specify allocation to treatment method); if randomization was used, please also state if restricted randomization was applied
- Indicate if masking was used during group allocation, data collection and/or data analysis

Please outline where this information can be found within the submission (e.g., sections or figure legends), or explain why this information doesn’t apply to your submission:

The study does not include experimental groups.

**Additional data files (“source data”)**

- We encourage you to upload relevant additional data files, such as numerical data that are represented as a graph in a figure, or as a summary table
- Where provided, these should be in the most useful format, and they can be uploaded as “Source data” files linked to a main figure or table
- Include model definition files including the full list of parameters used
- Include code used for data analysis (e.g., R, MatLab)
- Avoid stating that data files are “available upon request”

Please indicate the figures or tables for which source data files have been provided:

Data availability: The study comprises many different data sources. The PI does not have the legal right to share these data directly. UK Biobank data can be obtained from www.ukbiobank.ac.uk. The data repository for the Cambridge Centre for Ageing and Neuroscience (Cam-CAN) dataset can be found at [www.cam-can.org/index.php?content=dataset](http://www.cam-can.org/index.php?content=dataset). Access to BASE-II data can be obtained at [www.base2.mpg.de/7549/data-documentation](http://www.base2.mpg.de/7549/data-documentation). Access to VETSA data can be obtained at <https://medschool.ucsd.edu/som/psychiatry/research/VETSA/Researchers/Pages/default.aspx>. Betula is described at [www.umu.se/en/research/projects/betula---aging-memory-and-dementia/](http://www.umu.se/en/research/projects/betula---aging-memory-and-dementia/). For data from Barcelona brain studies, see [www.neurociencies.ub.edu/david-bartres-faz/](http://www.neurociencies.ub.edu/david-bartres-faz/). For LCBC Lifespan sample, contact information can be found at <https://www.oslobrains.no/presentation/anders-m-fjell/>. Part of the developmental sample can be accessed through <https://www.fhi.no/en/studies/moba/for-forskere-artikler/research-and-data-access/> (As of January 2021, we are in the process of transferring MRI data to this repository). Please note that for all samples, data transfer agreements must be signed and proper ethical and data protection approvals must be in place, according to national legislation. Code used for data analysis accompany the submission as separate files. The correlation matrices constituting the basis for the Mantel tests are also uploaded.

This info is provided in the manuscript under the section Data availability.
